# Establishment of time‐resolved fluoroimmunoassay of IgG4 based on magnetic microspheres

**DOI:** 10.1002/jcla.23874

**Published:** 2021-06-14

**Authors:** Qingqing Wu, Chen Hang, Ting Li, Binrong Wang, Yuan Qin, Yigang Wang, Xiumei Zhou, Pengguo Xia, Huiming Sheng, Pei Huang, Biao Huang

**Affiliations:** ^1^ College of Life Sciences and Medicine Zhejiang Sci‐Tech University Hangzhou China; ^2^ Tong Ren Hospital Shanghai Jiao Tong University School of Medicine Shanghai China; ^3^ College of Pharmacy Zhejiang Chinese Medical University Hangzhou China; ^4^ Department of Oncology Wuxi No. 2 Hospital Affiliated to Nanjing Medical University Wuxi China

**Keywords:** IgG4, IgG4‐RD, immunoassay, magnetic microspheres, time‐resolved fluorescence immunoassay

## Abstract

**Background:**

The abnormal increase in serum IgG4 level is an important clinical symptom of IgG4‐related disease (IgG4‐RD), and the detection of serum IgG4 level is a powerful tool for the diagnosis of IgG4‐RD. This study was conducted to establish a simple and rapid immunoassay for the determination of human serum IgG4 levels.

**Methods:**

Based on the competition method, a novel immunoassay was established for the determination of human serum IgG4 using a combination of time‐resolved fluoroimmunoassay (TRFIA) and magnetic microspheres. IgG4 was coupled with magnetic microspheres and competed with IgG4 in the samples to bind the Eu^3+^‐labeled anti‐IgG4 antibody. The immunocomplex was separated and washed in a magnetic field, and the fluorescence counts were measured according to the number of dissociated europium ions.

**Results:**

The analytical sensitivity of IgG4‐TRFIA based on magnetic microspheres was 0.006 g/L, and the detection range was 0.006–20 g/L under optimal conditions. The precision, recovery, and specificity of this immunoassay were demonstrated to be acceptable. The clinical application of IgG4‐TRFIA based on magnetic microspheres was evaluated and compared with that of immunonephelometry. The results showed that the two detection methods had a good correlation, with a correlation coefficient of .9871.

**Conclusion:**

IgG4‐TRFIA based on magnetic microspheres has the advantages of high sensitivity, wide detection range, and short analysis time and has the potential to become a useful tool for the diagnosis of IgG4‐RD.

## Graphical abstract

By the combination of time‐resolved fluoroimmunoassay (TRFIA) and magnetic microspheres, we established a simple and rapid immunoassay for the determination of human serum IgG4 levels. IgG4‐TRFIA based on magnetic microspheres has the advantages of high sensitivity, wide detection range, and short analysis time and has the potential to become a useful tool for the diagnosis of IgG4‐related disease (IgG4‐RD).

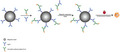

## 1 INTRODUCTION

Immunoglobulin G (IgG) is a type of immunoglobulin secreted by plasma cells; it has the highest content in serum (75%–80%). IgG can be divided into four subtypes according to its structure: IgG1, IgG2, IgG3, and IgG4. Different IgG subtypes have different contents and functions in the body. IgG4 accounts for 1%–7% of total IgG, and its content is the lowest among the four subtypes. Abnormal increases in serum IgG4 levels frequently occur in IgG4‐RD such as autoimmune pancreatitis (AIP), Mikulicz disease, and autoimmune hepatitis.^1^, ^2^, ^3^ Recently, a study^4^ proposed that IgG4‐RD can be divided into “proliferative” type and “fibrotic” type based on clinicopathologic characteristics. Most patients with “proliferative” type IgG4‐RD manifested high IgG4 levels, and the probability of detecting autoantibodies in these patients was higher.

IgG4‐RD is an immune‐mediated chronic fibrotic inflammatory disease that has been discovered in recent years. The disease was first proposed by Kamisawa et al.^5^ and was officially named in 2010.^6^ The disease is complex and can involve various organs and tissues, such as the pancreas, kidneys, lymph nodes, and thyroid. It is often accompanied by elevated serum IgG4 levels and IgG4‐positive cell infiltration of various organs.^7^, ^8^, ^9^, ^10^ The comprehensive diagnostic criteria for IgG4‐RD generally include clinical, serological, and histological examinations.^11^ The "International Consensus Guidelines for the Management and Treatment of IgG4‐related Diseases" suggests that the detection of serum IgG4 levels is an important tool for the diagnosis of IgG4‐RD, and a previous study^12^ suggested that serum IgG4 levels can serve as one of the serological indicators for early diagnosis of AIP. Moreover, dynamic monitoring of serum IgG4 levels may provide guidance for efficacy detection, recurrence prediction, and prognostic judgment in AIP. The detection of serum IgG4 levels is the most effective method for patients with relative contraindications for biopsy, as it can effectively exclude tumors or other diseases with clinical and pathological features similar to those of IgG4‐RD.^13^, ^14^ Therefore, the detection of serum IgG4 still has important clinical value; however, current traditional detection methods have certain limitations. Here, we propose a simple and rapid immunoassay method for the detection of serum IgG4 by combining the technical advantages of TRFIA and magnetic microspheres.

## 2 MATERIALS AND METHODS

### 2.1 Reagents and instruments

Human IgG4, anti‐human IgG4 monoclonal antibody, diethylenetriaminepentaacetic acid (DTPA), bovine serum albumin (BSA), Tris base, 1‐(3‐dimethylaminopropyl)‐3‐ethylcarbodiimide hydrochloride (EDC), and N‐hydroxysuccinimide (NHS) were purchased from Sigma‐Aldrich (St. Louis, MO, USA). Carboxyl‐modified magnetic microspheres were purchased from Suzhou Beaver Biomedical Engineering Co., Ltd. (Suzhou, China). The Sephadex‐G50 column was purchased from Seebio Biotech Co., Ltd. (Shanghai, China). Eu^3+^‐N^1^‐(p‐isothiocyanatobenzyl)‐diethylenetriamine‐N^1^, N^2^, N^3^, N^4^‐tetraacetic acid (DTTA), enhancement solution, MES buffer, the Eu^3+^ labeling kit, and the automatic time‐resolved immunofluorescence analyzer (TRF‐1000) were provided by Zhejiang Boshi Biotechnology Co., Ltd. (Hangzhou, China). The human IgG4 detection kit (immunonephelometry) and BN II fully automated protein analyzer were purchased from Siemens Healthcare Diagnostic Products (Marburg, Germany). All other chemicals were of analytical reagent grade.

### 2.2 Samples

Serum samples from 27 patients were provided by the Shanghai Tong Ren People's Hospital affiliated with Jiao Tong University. The study protocol was approved by the Medical Ethics Committee of Tong Ren Hospital, Shanghai Jiao Tong University School of Medicine (2020–35).

### 2.3 Preparation of IgG4‐coated magnetic microspheres

Thirty microliters of NHS (10 mg/ml) and 50 μl of EDC (10 mg/ml) were added to 10 mg of Fe_3_O_4_ microspheres, which had diameters of 2 μm, and the solution was mixed at 25℃ for 30 min. The mixture was magnetically separated and washed three times with 0.05 mol/L MES buffer (pH = 5.0) and was then suspended with the above buffer solution to make a magnetic particle suspension with a concentration of 100 mg/ml. Then, 1 ml of 0.05 mol/L MES buffer (pH = 5.0) and 50 μg of IgG4 were added to 100 μl of magnetic particle suspension, mixed well, and incubated overnight at 25℃. Then, the supernatant was removed after magnetic separation, and protein concentration was measured. The retained magnetic microspheres were washed three times with 0.05 mol/L Tris‐HCl buffer (containing 5% BSA, pH = 7.2) and blocked at 25℃ for 30 min. Finally, magnetic microspheres were washed three times with Tris‐HCl buffer (0.5% BSA, 0.1% NaN_3_, pH = 7.2), resuspended, aliquoted, and stored at 2–8℃.

### 2.4 Preparation and purification of IgG4 antibody labeled with Eu^3+^

Anti‐human IgG4 antibody labeling was performed following the instructions of the Eu^3+^ labeling kit. Anti‐IgG4 antibody (1 mg) was dissolved in 50 mmol/L Na_2_CO_3_‐NaHCO_3_ buffer (containing 0.155 mol/L NaCl, pH=8.5). Then, 0.2 mg of Eu^3+^‐N_2_‐[p‐isocyanate‐benzyl]‐DTTA (Eu^3+^‐DTTA) was added to 1 mg of anti‐IgG4 antibody solution, and the mixture was incubated with shaking at 25℃ for 20 h. The next day, the reaction solution was transferred to a Sephadex‐G50 column pre‐equilibrated with 80 mmol/L Tris‐HCl buffer (pH = 7.8) for purification. The fractions from the first peak containing the highest Eu^3+^ fluorescence counts were pooled and characterized. The anti‐human IgG4 antibody labeled with Eu^3+^ was diluted with elution buffer containing 0.2% BSA and stored at −20℃ until use.

### 2.5 Preparation of IgG4 standards

Different concentrations of IgG4 standard solutions (0.1, 0.5, 2, 5, and 20 g/L) were obtained by the dilution of high‐concentration IgG4 antigen in assay buffer (50 mmol/L Tris‐HCl buffer containing 8 mmol/L of NaCl, 0.1% BSA, 50 μmol/L of DTPA, 0.1 ml/L of Tween‐80, and 0.1% NaN_3,_ pH = 7.8), and assay buffer was used as the zero concentration point.

### 2.6 IgG4 detection procedure

**FIGURE 1:**
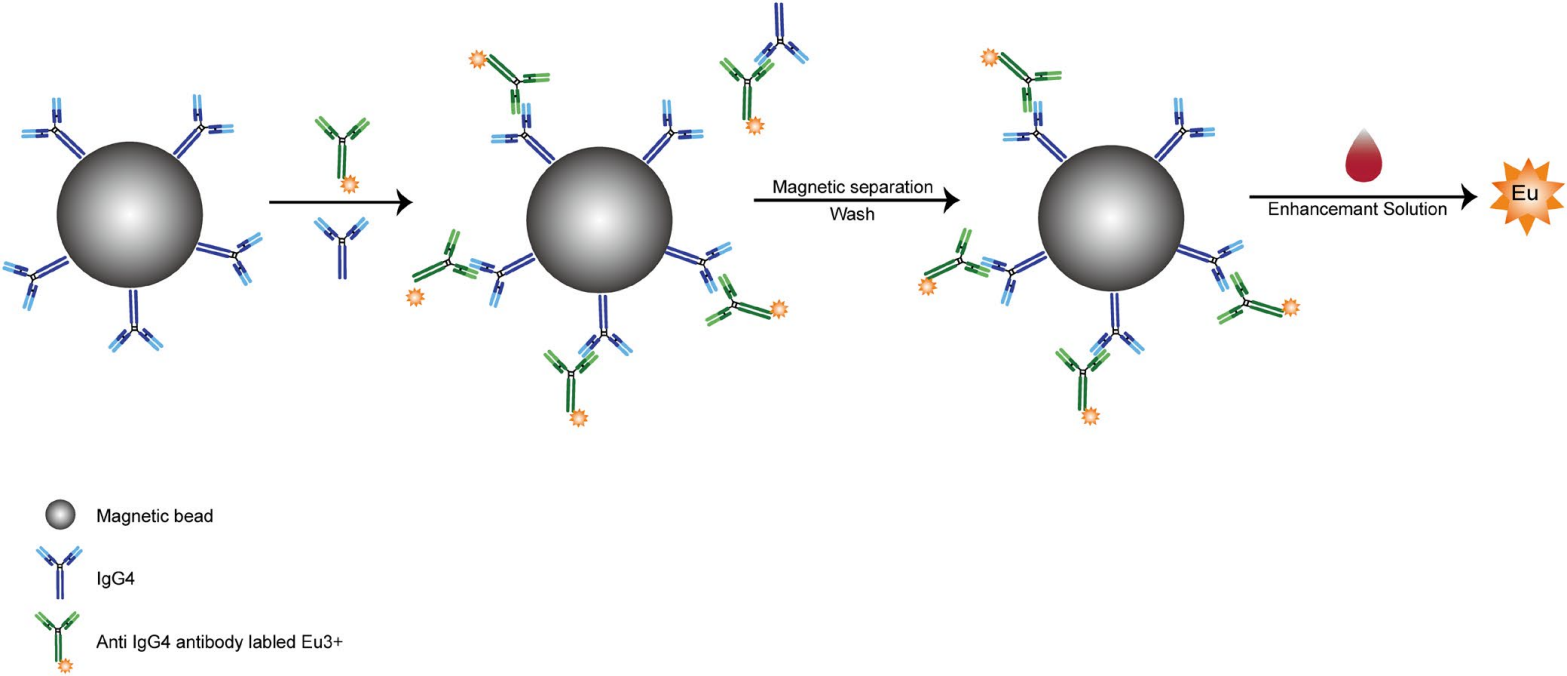
Schematic diagram of IgG4‐TRFIA based on magnetic microspheres

The levels of IgG4 in serum samples were determined based on the competition method (Figure 1). IgG4 standard solution (100 μl) or serum samples diluted with assay buffer (1:500), 50 μl IgG4‐coated magnetic microspheres, and 50 μl Eu^3+^‐labeled anti‐human IgG4 antibody diluted with assay buffer (1:100) were added to the reactive wells and incubated at 37℃ for 12 min. At this time, the magnetic particle‐IgG4‐Eu^3+^‐labeled anti‐human IgG4 antibody complex was formed. The mixture was magnetically separated and washed three times with washing buffer (50 mmol/L Tris‐HCl buffer containing 12.49 g/L NaCl and 1.11 g/L Tween‐20). Finally, 200 μl of the enhancement solution was added and reacted for 5 min at 25℃ with shaking. Fluorescence counts were measured using an automated time‐resolved immunofluorescence analyzer (TRF‐1000). The IgG4 concentration in the serum samples was determined according to the IgG4 standard curve.

### 2.7 Statistical analysis

Each serum sample was tested in duplicate. A commercial human IgG4 detection kit (immunoturbidimetry) was used to verify the clinical reliability of the established method, and the correlation between the two methods was evaluated using Pearson's correlation. ELISA Calc software (BioTNT, Shanghai, China) was used to perform log‐logit linear fitting for fluorescence counts (Y) and IgG4 standard concentration (X). SPSS version 19 (SPSS, Inc., Chicago, IL, USA) and GraphPad Prism 8.0 software (GraphPad, Inc., San Diego, CA, USA) were used for data analysis.

## 3 RESULTS

### 3.1 Optimization of the concentration of IgG4‐coated magnetic microspheres

**FIGURE 2:**
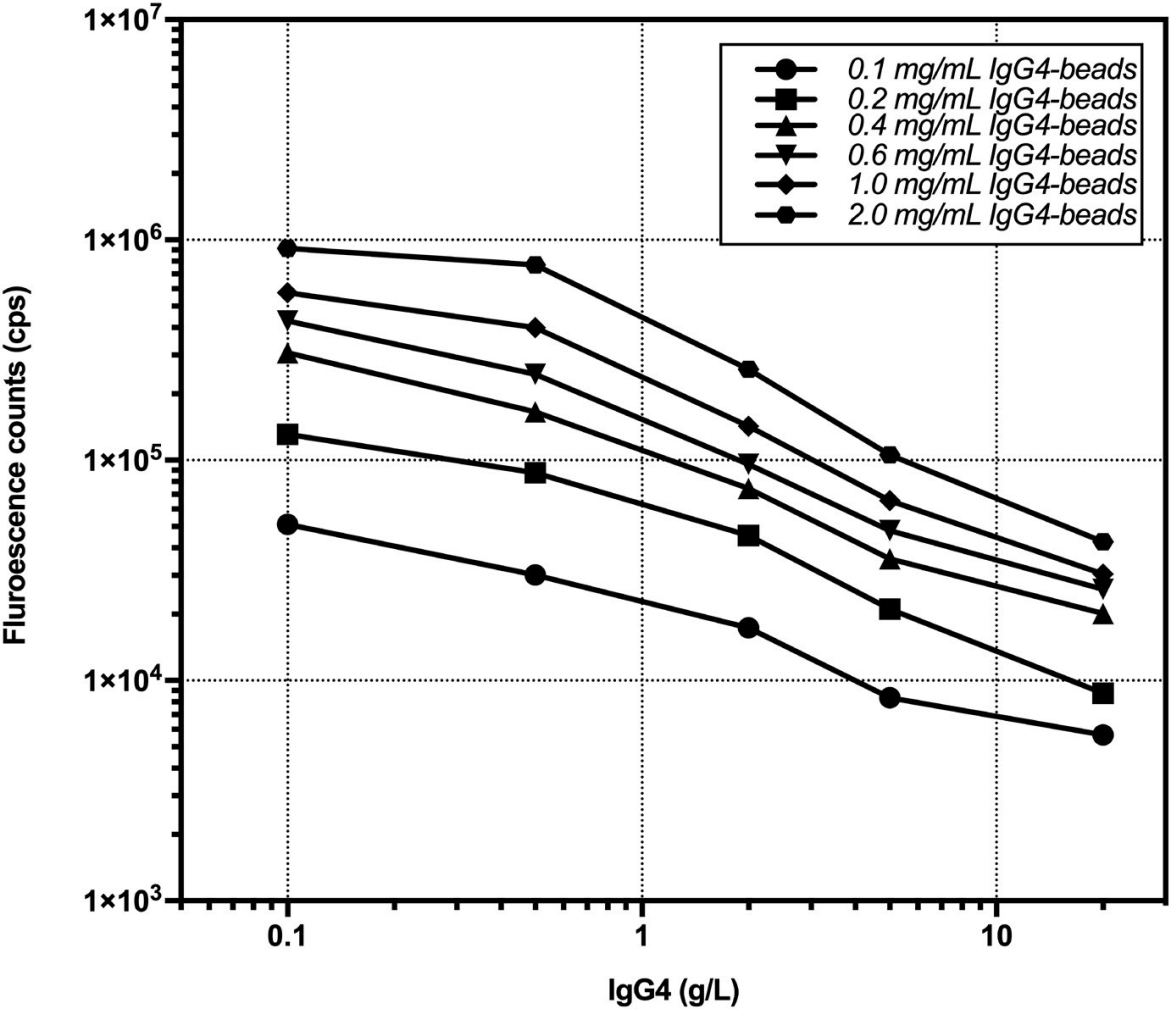
Optimization of the concentration of IgG4‐coated magnetic microspheres

Because detection was carried out on the fully automated TRF‐1000 time‐resolved immunofluorescence analyzer, the separation and washing times of the magnetic microspheres were fixed, and the effects of washing and separation were best when using the universal magnetic microspheres with diameters of 2 μm. Therefore, these microspheres were used for subsequent experiments. IgG4 (50 μg) was coupled with magnetic microspheres (10 mg). After magnetic separation, the protein content of the supernatant was 2.76 μg, which indicated that 47.24 μg of IgG4 had been coupled to magnetic microspheres, and the coupling rate was 94.48%, with an average of 4.72 μg of IgG4 coated per milligram of magnetic microspheres. IgG4 standard solution (100 μl) and Eu^3+^‐anti‐IgG4 antibody (50 μl) were added to 50 μl of different concentrations of IgG4‐coated magnetic microspheres, and the reaction mixture was incubated. The results showed that the detection range of this method was narrow when the concentration of IgG4‐coated magnetic microspheres was between 0.1 and 0.6 mg/ml, the sensitivity of this method was relatively low when the concentration was 2.0 mg/ml, and high sensitivity and a wide detection range could be obtained at 1.0 mg/ml (Figure 2).

### 3.2 Optimization of the concentration of Eu^3+^‐anti‐IgG4 antibody

**FIGURE 3:**
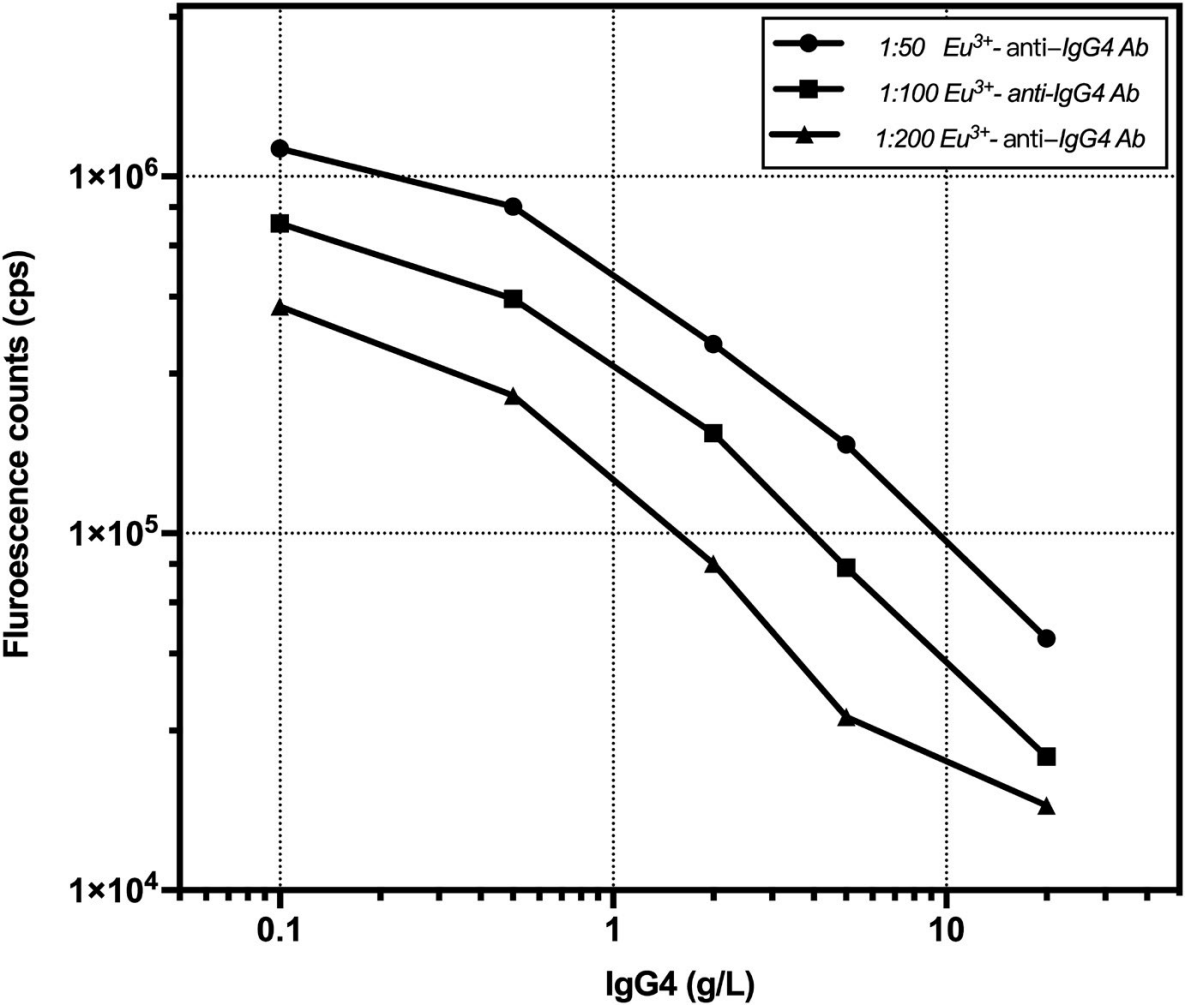
Optimization of the concentration of Eu^3+^‐anti‐IgG4 antibody

The optimal dilution of the Eu^3+^‐anti‐IgG4 antibody was determined at a high binding rate and low background. The purified Eu^3+^‐anti‐IgG4 antibody was detected at dilutions of 1:50, 1:100, and 1:200. When the Eu^3+^‐anti‐IgG4 antibody dilution was 1:100, the slope of the standard curve was the largest, thereby meeting the detection sensitivity and measurement range requirements (Figure 3).

### 3.3 Analytical sensitivity and detection range

**FIGURE 4:**
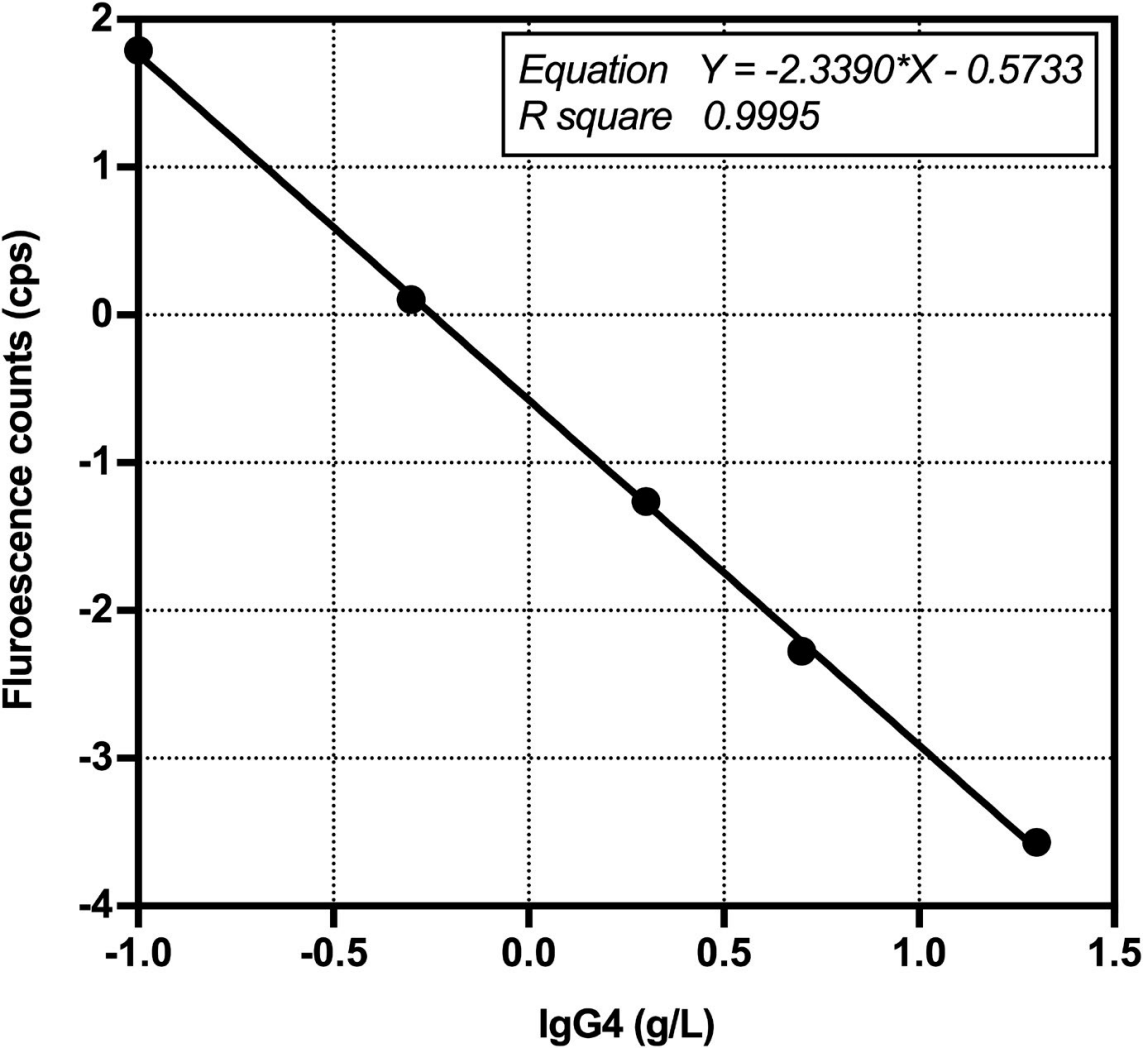
Standard curve of IgG4‐TRFIA

The standard curve was obtained by repeating the detection of IgG4 standard solutions at different concentrations (0, 0.1, 0.5, 2, 5, and 20 g/L) twice. Since the assay method in this study was established based on the competition method, linear fitting could be performed using log‐logit (Figure 4). Under optimal conditions, the equation of the standard curve was expressed as Y = −2.3390X−0.5733, and the correlation coefficient was .9995. The analytical sensitivity of IgG4‐TRFIA was 0.006 g/L (defined as the concentration value corresponding to the mean value minus two times the standard deviation [n = 10]).

### 3.4 Precision and recovery

The accuracy of the established method was evaluated using the intra‐ and inter‐assay coefficients of variation (CV). Three quality‐controlled samples with different concentrations (low, medium, and high) were detected, 10 wells were set for each quality‐controlled sample, and the experiment was repeated three times. The range of intra‐assay CV was 3.70–5.09%. The range of inter‐assay CV was 4.89–6.26%, and the intra‐ and inter‐assay CVs of each quality‐controlled sample were ≤10%. The recovery rate was evaluated by adding a high‐concentration IgG4 standard to samples of known concentration at a ratio of 1:9. The recovery rate was calculated as recovery rate (%) = (measured concentration/theoretical concentration) × 100%. The range of recovery rates was 99.39–107.90%, indicating that the precision of this method was good. Because this assay method involved a large‐ratio dilution of samples, in order to evaluate the matrix effect of assay buffer on serum, a serum sample containing a high concentration of IgG4 (15.82 g/L) was diluted with assay buffer at different dilution ratios for detection, and the difference between the expected and measured concentrations was within 10.00%, indicating that the matrix effect of assay buffer met the requirements (Table 1).

**TABLE 1:** Matrix effect in serum samples

Samples	Dilution ratio	Expected (g/L)	Measured (g/L)	Recovery (%)
IgG4 (g/L)	1/2	7.91	8.28	104.68
	1/4	3.95	3.69	93.42
	1/8	1.97	1.89	95.94
	1/16	0.99	0.96	96.97

### 3.5 Specificity

The specificity of the assay method was evaluated by detecting four possible interfering substances: IgM, IgG1, IgG2, and IgG3. The results showed that the measured concentrations of IgM, IgG1, IgG2, and IgG3 were between 0.01–0.03 g/L, indicating that IgG4‐TRFIA based on magnetic microspheres has high specificity for IgG4 and can be used for the clinical detection of serum IgG4.

### 3.6 Clinical applications of IgG4‐TRFIA based on magnetic microspheres

**FIGURE 5:**
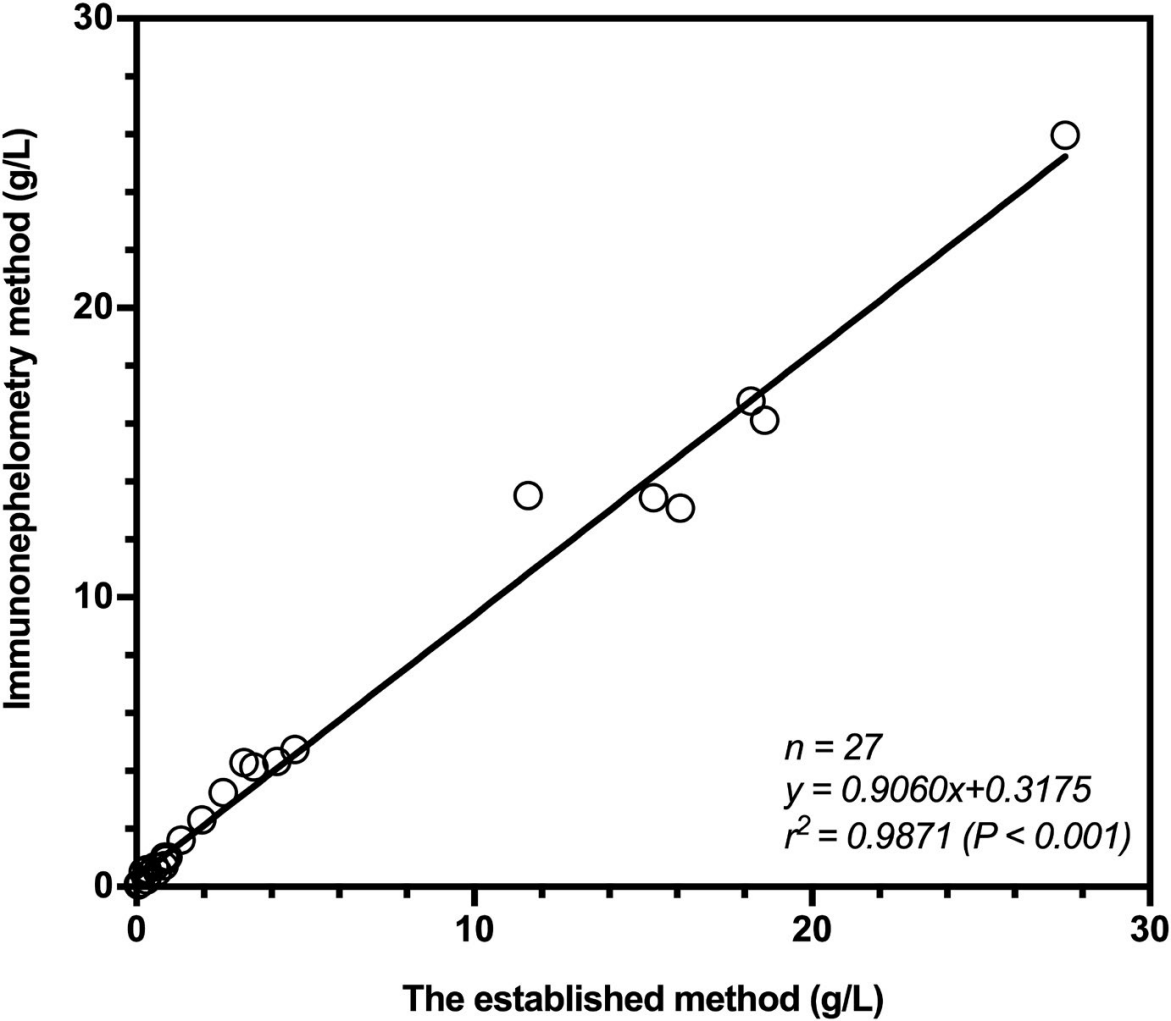
Comparison between the established method and immunonephelometry method

To further evaluate the clinical application value of the immunoassay method established for detection of human serum IgG4, this method and the human IgG4 detection kit (immunonephelometry) were used to detect IgG4 in 27 serum samples. The results showed that the two methods were in good agreement, with a correlation coefficient of r^2^ = .9871 (Figure 5), indicating that IgG4‐TRFIA based on magnetic microspheres can be used for the clinical detection of human serum IgG4.

## 4 DISCUSSION

To date, a variety of assay methods have been developed for the detection of serum IgG4 levels, including radio immunodiffusion (RID),^15^ enzyme‐linked immunosorbent assay (ELISA),^16^ immunonephelometry,^17^ and other methods. In addition, a previous study described a method based on IgG subclass‐specific tryptic peptides and liquid chromatography tandem‐mass spectrometry (LC‐MS/MS) for quantification of IgG subclasses.^18^ This method can avoid antigen excess, and the analytical measurement range is similar to that of immunonephelometry. However, complicated pre‐processing of samples, such as serum or plasma, is usually required to perform LC‐MS/MS analyses and requires experienced technicians for operators. RID is the earliest assay method used to detect serum IgG4, but is seldom used now due to the high consumption of serum, long detection times (48–72 h), and low sensitivities involved. ELISA can be used for qualitative or semi‐quantitative analysis; however, the influencing factors of ELISA vary, reagent stabilities are poor, and reaction times are relatively long (4 h). Currently, serum IgG4 levels are routinely quantified using immunonephelometry. Compared with RID and ELISA, immunonephelometry has the advantages of automatic operation, good accuracy, and reproducibility; however, clinical use is limited because of the relatively high instrument costs involved. In addition, in some cases this method is not very effective. For example, at present, the detection range of the human IgG4 determination kit is 0.052–3.3 g/L, and the detection range is relatively narrow. The serum IgG4 level in most patients with IgG4‐RD is higher than 3.3 g/L; therefore, the serum sample needs to be diluted 1:2000 to make the concentration within the range of the calibration curve. Such a large‐ratio dilution will increase detection error and the time required to produce results. On the other hand, due to the presence of a large number of complexes and proteins in serum samples, these interfering substances produce non‐specific scattered light, which affects the sensitivity of detection. In contrast, the assay method for serum IgG4 established in this study has higher sensitivity (0.006 g/L) and a wider linear range (0.006–20 g/L), which allows repeated sample dilutions to be avoided. Furthermore, the assay method has high specificity and good stability. This is due to the unique technical advantages of TRFIA and magnetic microspheres.

TRFIA is an emerging technology that uses lanthanide chelates as a marker. Advantages of TRFIA include zero background, wide linear range, and good stability, and it is widely used in the diagnosis of autoimmune diseases.^19^, ^20^, ^21^ Lanthanide chelates have small molecular weights, high fluorescence intensities, and good stabilities; therefore, the three‐dimensional structures and stabilities of the labeled substance are not affected, and the immunoassay method established has high sensitivity, good repeatability, and a wider linear range. Lanthanide chelates also exhibit unique fluorescence characteristics.^22^ Compared with ordinary fluorophores, the Stokes shift of lanthanides is exceptionally large (especially Eu^3+^ chelates, which can reach 290 nm), which makes it easy to separate excitation and emission wavelengths. Moreover, this helps to eliminate interference from non‐specific fluorescence, thereby enhancing the specificity of detection. A delay time can be introduced after the samples are excited by pulsed light, and the fluorescence counts are measured only after the short‐lived background fluorescence caused by serum, solvents, and reaction wells is completely quenched. Thus, the sensitivity of detection is improved, and a high signal‐to‐noise ratio is achieved.

The excellent analytical performance and reduced reagent costs of TRFIA make it an attractive method for serum IgG4 detection. However, the limitations of the conventional TRFIA remain. For example, antigens or antibodies are usually immobilized, and the contact area with the target substance is small, which increases the reaction time needed (1 h). The magnetic microspheres, an important tool in immunoassays, uniformly and stably distributed in the reaction solution to provide a larger binding surface area and could bind many Eu^3+^‐labeled anti‐IgG4 antibodies in a short time. This improved the analysis sensitivity, and the reaction time was significantly shortened (to 12 min). As IgG4 is coupled with magnetic microspheres by chemical groups (the coupling rate was 94.48%), the loss of IgG4 due to washing is reduced, and the precision of detection is improved.

In conclusion, we established a novel immunoassay method for the detection of human serum IgG4. This method combines the advantages of TRFIA and magnetic microspheres and has the advantages of simple operation, short detection times, wide detection ranges, and high sensitivities. Moreover, the method established has good agreement with immunonephelometry and provides a useful new technique for the detection of human serum IgG4.

## CONFLICTS OF INTEREST

The authors declare no conflicts of interest.

## AUTHOR CONTRIBUTIONS

Q.W., C.H., T.L., M.S., P.H., and B.H. designed the experiments; Q.W., C.H., T.L., and R.W. performed most of the experiments; Q.W., C.H., T.L., and R.W. analyzed experimental data; Q.W. and C.H. drafted the manuscript; Y.Q., G.W., M.Z., P.X., M.S., P.H., and B.H. revised the manuscript. All authors contributed to the manuscript revision, read, and approved the submitted version.

## ETHICAL STATEMENT

The study protocol was approved by the Medical Ethics Committee of Tong Ren Hospital, Shanghai Jiao Tong University School of Medicine (2020–35).

## Data Availability

All the data that related to this study are available from the corresponding author upon reasonable request.
